# Divergent Patterns in Care Utilization and Financial Distress between Patients with Blood Cancers and Solid Tumors: A National Health Interview Survey Study, 2014–2020

**DOI:** 10.3390/cancers14071605

**Published:** 2022-03-22

**Authors:** Christopher T. Su, Christine M. Veenstra, Minal R. Patel

**Affiliations:** 1 Institute for Healthcare Policy and Innovation, University of Michigan, Ann Arbor, MI 48109, USA; cveenstr@med.umich.edu (C.M.V.); minalrp@umich.edu (M.R.P.); 2 Division of Hematology and Oncology, Department of Internal Medicine, University of Michigan, Ann Arbor, MI 48109, USA; 3 Rogel Cancer Center, Michigan Medicine, Ann Arbor, MI 48109, USA; 4 Department of Health Behavior & Health Education, School of Public Health, University of Michigan, Ann Arbor, MI 48109, USA

**Keywords:** health services research, financial hardship, financial stress, healthcare surveys, health services accessibility, hematologic neoplasms, medical oncology

## Abstract

**Simple Summary:**

Patients with blood cancers and solid tumors embark on very different journeys when receiving cancer care. We sought to better understand care patterns and financial barriers experienced by the two groups by using a large national survey. We found respondents with blood cancers attended more medical visits and were more worried about paying medical bills compared to those with solid tumors. However, they were less likely to delay medical care due to cost. Our results showed that patients and survivors with blood cancers should be recognized as a distinct group in future research to best tailor interventions to their unique needs.

**Abstract:**

Introduction: Important differences exist between the presentation, treatment, and survivorship of patients and survivors with blood cancers. Furthermore, existing research in financial toxicity has not fully addressed the relationship between medical care utilization and patient-reported outcomes of financial barriers and distress. We answered these questions by using a nationally representative survey. Methods: Respondents with blood cancers and solid tumors from the National Health Interview Survey were identified (2014–2020). We identified 23 survey questions as study outcomes and grouped them into three domains of medical care utilization, financial barriers to care, and financial distress. Associations between the three domains and associations of study outcomes between cancer types were examined using weighted univariate analyses and multivariable linear and logistic regressions. Results: The final study group consisted of 6248 respondents with solid tumors and 398 with blood cancers (diagnosed ≤ 5 years). Across all respondents with cancer, higher medical care utilization is generally associated with increased financial barriers to care. Compared to respondents with solid tumors, respondents with blood cancers had a higher level of medical care utilization (β = 0.36, *p* = 0.02), a lower level of financial barriers to care (β = −0.19, *p* < 0.0001), and a higher level of financial distress in affording care (β = 0.64, *p* = 0.03). Conclusions: Patients and survivors with blood cancers and solid tumors demonstrate divergent patterns in care utilization, financial barriers, and financial distress. Future research and interventions on financial toxicity should be tailored for individual cancer groups, recognizing the differences in medical care utilization, which affect the experienced financial barriers.

## 1. Introduction

Financial toxicity, an adverse effect experienced by patients due to high and often unsustainable cost of medical care, is increasingly recognized and targeted for interventions [1,2,3,4,5,6,7,8,9]. Financial toxicity is multifaceted and comprises both objective financial burden (including drug costs, related medical costs, and indirect costs, such as lost work productivity) as well as subjective financial distress [1,3,9]. Due to its complexity, financial toxicity must be measured and interventions designed in view of multiple levels of the healthcare system [4,10,11,12]. Importantly, financial toxicity is only incompletely assessed by using all-claims out-of-pocket copayment data, as these databases do not capture the extent of financial barriers experienced by the patient. Thus, studies utilizing these databases [13,14,15] provide an incomplete picture of financial toxicity. On the other hand, large national databases, such as the Surveillance, Epidemiology, and End Results Program (SEER) contain detailed cancer-specific diagnostic and treatment information but no care utilization or financial data. Therefore, due to the limitations of large national databases, financial toxicity has generally been studied at the level of individual cancer centers; however, these studies may not be nationally representative [16,17,18]. To overcome these challenges, we employed the National Health Interview Survey (NHIS), which contains both patient-reported cancer diagnoses and measures of financial difficulty and financial distress. Investigators had recognized this advantage and leveraged NHIS to study financial barriers faced by cancer survivors in other studies [19,20,21,22].

We raise two unanswered questions in the existing literature. First, the relationship between medical care utilization (broadly defined as inpatient and outpatient visits) and patient-reported financial barriers and distress is not well defined. Although the amount of medical care utilization for selected cancer populations has been studied in all-claims databases [14,23,24], they do not contain any patient-reported financial difficulty. As NHIS contains both of these elements, we studied the association between patient-reported medical care utilization with financial barriers and distress. We hypothesize that higher levels of medical care utilization will lead to more financial barriers and financial distress. Second, patients with blood cancers and solid tumors are not the same. Although blood cancers only comprise approximately 10% of all cancers [25], significant differences exist in the clinical course, treatment options, and overall morbidity and mortality between blood cancers and solid tumors [26,27,28,29,30,31]. NHIS distinguishes between blood cancer and solid tumors in patient-reported cancer diagnoses, and comparisons can be made between reported medical care utilization with financial barriers and distress in each group. Although we recognize the heterogeneity in clinical course and treatment options among the blood cancers, there is an even wider degree of differences when all cancers are examined as a large group in prior studies of financial toxicity [3,9,11,12]. Especially with overall survival rapidly improving in blood cancers, with many patients entering a prolonged survivorship period [32,33], a detailed examination of the differences in medical care utilization, financial barriers of affording care, and financial distress between cancer types is warranted. We hypothesize that respondents of blood cancers experience higher medical care utilization, financial barriers, and financial distress compared to those with solid tumors.

## 2. Methods

### 2.1. Cohort Identification

NHIS is an annual, nationally representative, cross-sectional household survey of the civilian United States adult population conducted by the National Center of Health Statistics, a branch of the Centers for Disease Control and Prevention. The primary objective of NHIS is to serve as an annual barometer of trends in illness and medical care via the collection of a wide spectrum of patient-reported measures of health. NHIS is not a longitudinal survey, and participants are randomly selected using household addresses each year; each survey takes approximately one hour to complete [34]. We obtained NHIS data for 2014–2020, collated by the Integrated Public Use Microdata Series (https://www.nhis.ipums.org (accessed on 10 January 2022)) [35]. We identified NHIS respondents with a self-reported prior cancer diagnosis within 5 years of completing the NHIS survey and stratified them into two groups comprising respondents with blood cancers (with a prior diagnosis of leukemia, lymphoma, or blood cancer) and respondents with solid tumors (24 different types of cancer; see Supplementary Table S1). We selected survey data after 2014 due to the implementation of the Affordable Care Act, which affected healthcare cost for individuals with cancer significantly [19,22,36]. Self-reported baseline demographics were extracted from the survey data. As NHIS is a fully de-identified database per federal law, with no publicly accessible private health information, this study is exempt from institutional review board review.

### 2.2. Study Measures

Our primary dependent variables of interest were categorized into three domains: medical care utilization in the last 12 months, financial barriers to care in the last 12 months, and financial distress of affording care. Each domain comprises a series of NHIS survey questions, which were categorized by the investigators following exhaustive review of the NHIS codebook; the full text of survey questions and other data are listed in Supplementary Table S2. Factor analysis was employed for each domain to further condense the study outcomes. Retained factors were determined by using a minimum eigenvalue of 1.0 (Kaiser criterion) and visual confirmation of the scree plot to confirm the validity of the Kaiser criterion in retaining factors with an eigenvalue >1.0.

#### 2.2.1. Medical Care Utilization in the Last 12 Months

We identified 6 questions that assessed medical care utilization in the last 12 months, including inpatient hospitalizations, emergency room visits, and outpatient clinic visits.

#### 2.2.2. Financial Barriers to Care in the Last 12 Months

We identified 9 questions that assessed financial barriers to care in the last 12 months, including the deferral or delay of necessary medical care or medications due to concerns over cost. 

#### 2.2.3. Financial Distress of Affording Care

We identified 8 questions that assessed financial distress of affording medical care, including difficulty in paying medical bills and financial worry regarding care costs.

Independent variables chosen by investigators via a review of demographic, financial, and health variables contained in the NHIS codebook included self-reported age, sex, race, ethnicity, marital status, number of persons in the family, educational status, total combined family income, health insurance status, baseline health status, physical activity limitation, and geographic region. 

### 2.3. Statistical Analysis

Univariate analyses were performed to assess differences in baseline demographics between cancer types. To determine differences between patients with solid tumors and blood cancers, associations between the extracted factors of each domain with cancer type were examined using multivariable linear regression, controlling for baseline characteristics, which were significant in univariate analysis of independent variables (sex, total family income, and baseline health status). Multivariable regressions were performed to examine the relationship between medical care utilization with financial barriers and financial distress and between the study outcomes and cancer type.

Univariate analyses were performed via Pearson’s chi-squared tests with Rao–Scott correction and two-sample differences of means *t*-test for categorical and continuous variables, respectively. Multivariable analyses were performed using linear or logistic regression. All analyses were weighted and stratified based on IPUMS/NHIS study design [37]. We defined a *p* value of <0.05 as statistically significant. Statistical analyses were performed using SAS (version 9.4, Cary, NC, USA). 

## 3. Results

### 3.1. Cohort Identification

**Figure 1:**
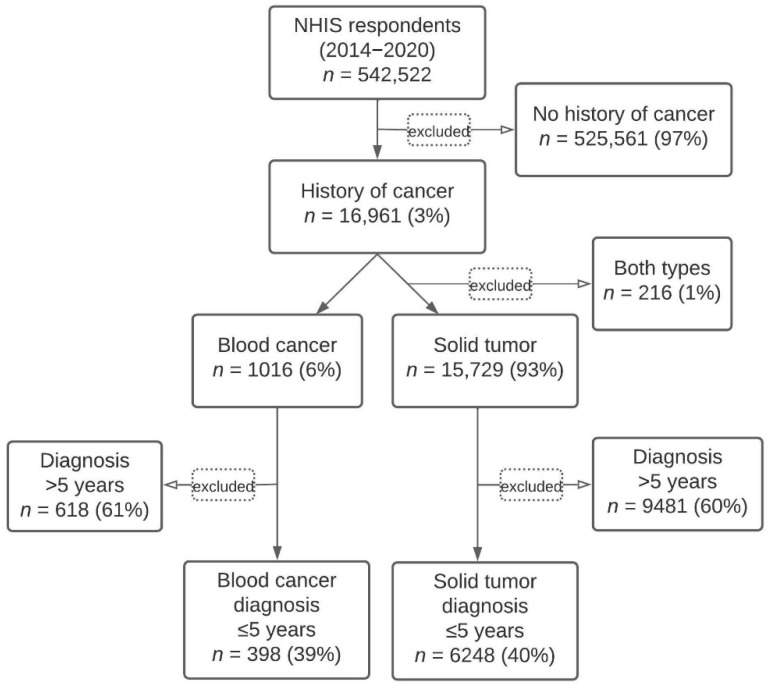
NHIS cohort identification.

The NHIS cohort identification schematic is shown in Figure 1. From 2014 to 2020, 16,961 (3%) of NHIS respondents self-reported a diagnosis of cancer, with 15,729 (93%) being solid tumor and 1016 (6%) being blood cancer. Respondents with a history of both blood cancers and solid tumors (*n* = 216) were excluded from the analysis. The respondents were further stratified by time from diagnosis, with the final cohorts comprising 6248 respondents with solid tumors and 398 with blood cancers who were diagnosed within the last 5 years. In the blood cancer group, approximately 56% of respondents had lymphoma, 30% leukemia, and 13% other blood cancers. In the solid tumor group, approximately 22% had breast cancer, 17% prostate cancer, 9% melanoma, and 11% had more than one type of solid tumor (Supplementary Table S1). 

### 3.2. Respondent Characteristics

Respondent characteristics, including income, health insurance, and baseline health status, are shown in Table 1. More respondents with blood cancers were male (52.8 vs. 44.7%, *p* = 0.01) and had higher total combined family income (*p* = 0.03) compared to those of respondents with solid tumors. Mean time since diagnosis was 1.5 years for respondents with blood cancers and 2.2 for respondents with solid tumors. There were no significant differences in insurance status between groups. Respondents with blood cancers self-reported poorer baseline health (40.6% vs. 32.3%, *p* < 0.01).

### 3.3. Factor Analysis

Following factor analysis performed on study outcomes within each domain, one representative factor was extracted for each domain following application of the Kaiser criterion (Supplementary Figure S1 and Table 2). For the extracted factor of the medical care utilization domain (eigenvalue 1.30), the study outcome with the highest factor loading (strength of correlation of the study measure to the extracted representative factor for each domain) was “received care 10+ times” (0.59). For the extracted factor of the financial barriers to care domain (eigenvalue 4.16), the study outcomes with the highest factor loading were “delayed refilling medications to save money” (0.80) and “took less medication to save money” (0.80). For the extracted factor of the financial distress of affording care domain (eigenvalue 4.41), the study outcome with the highest factor loading was “worried about monthly bills” (0.84).

### 3.4. Association of Medical Care Utilization with Financial Barriers and Financial Distress

**Figure 2:**
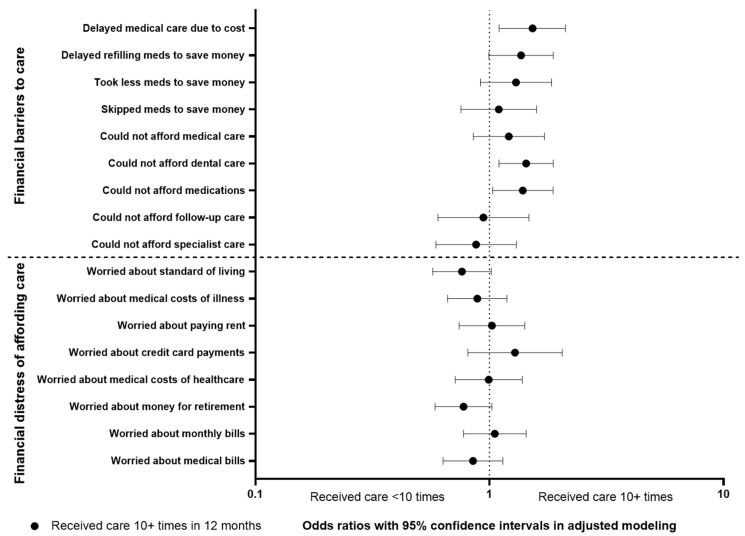
Multivariable analyses of association between medical care utilization (represented by receiving care 10+ times in 12 months) and study outcomes in the domains of financial barriers to care and financial distress of affording care.

The variable “received care 10+ times” was selected as the representative variable for medical care utilization, as it was the study outcome with the highest factor loading in the extracted factor for the domain. Multivariable analysis of its association with study outcomes in the financial barrier and financial distress domains revealed that increased medical care utilization is associated with a general trend in increased financial barriers across all study respondents, but there is no clear association with financial distress (Figure 2). The analysis revealed significant associations of higher medical care utilization and delaying medical care due to cost (odds ratio (OR) with 95% confidence interval 1.529 (1.100–2.110), *p* < 0.01), being unable to afford dental care (OR 1.435 (1.099–1.874), *p* < 0.01), and being unable to afford medications (OR 1.388 (1.031–1.870), *p* = 0.03). All of these outcomes are in the financial barriers to care domain.

### 3.5. Associations of Cancer Type with Study Outcomes

#### 3.5.1. Extracted Domain Factors

Multivariable regression analysis between the extracted factors of each domain and cancer type revealed a higher level of medical care utilization (β = 0.36, *p* = 0.02), a lower level of financial barriers to care (β = −0.19, *p* < 0.0001), and a higher level of financial distress in affording care (β = 0.64, *p* = 0.03) in respondents with blood cancer (Table 3A). 

#### 3.5.2. Multivariable Analyses of Study Outcomes

In the domain of medical care utilization, multivariable analyses (Table 3B) revealed respondents with blood cancer were more likely to see or speak to a medical specialist compared to those with solid tumors (OR 1.73 (1.17–2.57), *p* = 0.01). 

In the domain of financial barriers to care, multivariable analyses (Table 3B) revealed respondents with blood cancer were less likely to delay refilling medications to save money (OR 0.37 (0.18–0.76), *p* = 0.01), take less medications to save money (OR 0.42 (0.20–0.86), *p* = 0.02), be unable to afford medications (OR 0.51 (0.27–0.96), *p* = 0.04), be unable to afford follow-up care (OR 0.29 (0.10–0.89), *p* = 0.03), and be unable to afford specialist care (OR 0.22 (0.07–0.73), *p* = 0.01). 

In the domain of financial distress of affording care, multivariable analyses (Table 3B) revealed respondents with blood cancers were more likely to worry about the medical costs of healthcare (OR 3.36 (1.50–7.51), *p* < 0.01).

## 4. Discussion

Due to the lack of patient-reported financial toxicity measures in all-claims databases [13,14,15,23,24] and SEER, we utilized NHIS to examine the association between medical care utilization and cancer type with patient-reported financial barriers and financial distress. We found that increased medical care utilization is associated with increased financial barriers, but no significant associations were seen with financial distress. In addition, we found that respondents with blood cancers reported increased medical utilization and distress over the medical costs of healthcare and paying medical bills compared to those with solid tumors. However, respondents with blood cancers were less likely to modify their medical care or medication use as a response to the cost of care.

Previous studies have reported high levels of medical care utilization in cancer patients [7,28], especially those with blood cancers [23,24,28,38]. However, these studies have not been able to directly associate medical care utilization with patient-reported measures of financial barriers and financial distress, which is possible in NHIS. Our analysis among all respondents with cancer demonstrated that there are significant associations between higher medical care utilization and higher financial barriers to care, including delaying medical care and being unable to afford medications.

Examining the difference between blood cancers and solid tumors specifically, existing studies have demonstrated that the clinical course, treatment options, and overall morbidity and mortality among the two types of cancers are fundamentally different [26,27,28,29,30,31]. These differences have significant implications for the financial toxicity experienced by patients and survivors. We found that respondents with blood cancers reported increased medical utilization and distress over the medical costs of healthcare and paying medical bills compared to those with solid tumors. The high costs associated with providing care to patients with blood cancers are well documented [23,26,28,39]. Our study corroborates these findings, as respondents with blood cancers reported a higher level of medical care utilization, especially in the domains of length and frequency of hospitalizations, as well as the number of outpatient visits. Thus, we questioned whether this level of increased medical care utilization then translated into tangible financial barriers.

Surprisingly, we found respondents with blood cancers were less likely to experience financial barriers to medical care or modify their care as a response to cost across almost all assessed outcomes in this domain, despite apparent higher care utilization. We had hypothesized that increased medical care utilization will lead to more out-of-pocket expenses and subsequent modification of care, as also shown in our analysis of medical care utilization in all respondents with cancer. Consequently, we suspect that differences in inpatient versus outpatient site of care, treatment course, and disease morbidity in our NHIS sample contributed to this finding. Previous studies have described differences in clinical morbidity and outcomes of patients with blood cancers and found that patients with blood cancers were more likely to die after hospitalization with COVID-19 [40,41] and were more likely to die in the hospital and receive intensive care compared to patients with solid tumors [30,31,42]. Although limited in scope of analysis, these studies point to higher baseline morbidity and need for inpatient care of patients with blood cancers. Thus, we theorize that these differences, especially compounded with a shorter survivorship period compared to many types of solid tumors [25,43], may explain the decreased level of reported financial barriers observed in our NHIS sample.

Nonetheless, these findings call for recognition of the unique treatment and survivorship journey of patients with blood cancers and the need for dedicated research. As financial toxicity research in patients with cancer moves into the realm of interventions [6,10,44,45], it is important to consider the drivers of that financial toxicity. Given the high levels of care utilization seen in the respondents with blood cancer in our study, we question whether medical care utilization is the most prominent driver of financial barriers and distress. Thus, interventions should consider the out-of-pocket costs and lost opportunity costs associated with high levels of inpatient admissions and outpatient visits, which is present in blood cancers but also common to specific types of solid tumors. Given the rapid increase in overall survival and migration of blood cancer care into the outpatient setting [46,47], more research is presently needed to clarify the specific financial barriers faced by blood cancer patients. 

The most important limitation of our study is patient-reported cancer diagnosis and the lack of corroborating cancer diagnostic or treatment data, which is a limitation common to all NHIS cancer studies [19,21,22,48]. Similarly, NHIS only includes adults, medical comorbidity data is sparse, and categorization of cancer types is limited due to survey design. However, we consider the inclusion of patient-reported financial barrier and distress measures an important strength that mitigates this limitation, as this is unique to NHIS among the large databases. Second, our study parameters include NHIS respondents diagnosed with cancer from 2009 to 2020. Given the rapid advances in cancer treatment, this extended timeframe could affect our examined study outcomes; however, the larger sample size available in a nationally representative survey sample provides valuable insight not present in single-center studies. Third, it was difficult to fully adjust for the effects of insurance in our models due to data limitations, although the two cancer types did not show a baseline difference when stratified by several insurance categories. Finally, assessed medical care covers all healthcare visits and is not specific to cancer-related care. However, we expect a large amount of this higher medical care utilization to be related to cancer care [7,28,49].

## 5. Conclusions

We examined the relationship between medical care utilization with financial barriers to care and financial distress of affording care in NHIS among respondents with cancer and found that increased medical care utilization is significantly associated with increased financial barriers to care. We also specifically examined these study outcomes comparing respondents with blood cancers and solid tumors. We found that respondents with blood cancers reported significantly more medical care utilization and financial distress compared to their counterparts with solid tumors. However, they were less likely to delay or report the inability to afford care, with the exact reason to be clarified in future research. Due to these important differences across cancer types, which might be attributed to differing medical care utilization, patients and survivors with blood cancers should be recognized as a distinct group apart from those with solid cancers in financial toxicity research.

## Figures and Tables

**Table 1:** Respondent demographics.

Baseline Demographic Variables	Respondents with Cancer		
	Blood Cancer Respondents (95% Confidence Interval)	Solid Tumor Respondents (95% Confidence Interval)	*p* Value
N	398	6248	
Mean age, years	61.5 (59.2–63.7)	63.5 (63.0–64.0)	0.08
Mean time after diagnosis, years	1.5 (0.8–2.2)	2.2 (1.9–2.4)	0.08
Sex, male (%)	52.8 (46.5–59.1)	44.7 (43.1–46.2)	0.01
Race, white (%)	93.4 (90.5–96.3)	90.2 (89.2–91.2)	0.08
Ethnicity, Hispanic (%)	6.3 (3.4–9.2)	8.9 (7.7–10.1)	0.15
Marital status, married (%)	59.7 (53.6–65.7)	58.5 (57.0–60.1)	0.72
Persons in the family, number	2.4 (2.3–2.6)	2.3 (2.2–2.3)	0.06
Educational status, college and above (%)	64.8 (57.5–72.0)	61.0 (59.1–62.9)	0.33
Total combined family income (%)			0.03
Less than USD 50,000	37.7 (31.8–43.6)	46.6 (44.9–48.4)	
USD 50,000–USD 99,999	33.0 (27.0–39.0)	28.7 (27.1–30.2)	
USD 100,000 or more	29.3 (22.9–35.7)	24.7 (23.2–26.2)	
Above the poverty threshold (%)	92.8 (90.0–95.6)	89.6 (88.5–90.7)	0.07
Currently lacks health insurance coverage (%)	2.4 (0.1–4.8)	3.2 (2.6–3.8)	0.56
Currently covered by Medicaid or other public assistance/state-sponsored plan (%)	14.3 (9.2–19.4)	15.2 (13.8–16.6)	0.75
Currently covered by private health insurance (%)	64.0 (58.4–69.6)	59.1 (57.4–60.7)	0.10
Currently covered by Medicaid (%)	10.7 (7.3–14.1)	12.3 (11.2–13.4)	0.40
Currently covered by Medicare (%)	52.8 (46.4–59.3)	55.6 (53.9–57.2)	0.42
Baseline health status, fair to poor (%)	40.6 (34.3–46.9)	32.3 (30.8–33.7)	0.008
Activity limitation (%)	62.3 (54.6–70.0)	61.7 (59.8–63.6)	0.89
Geographic region (%)			0.13
Northeast	21.5 (16.3–26.8)	19.5 (17.9–21.0)	
Northcentral/Midwest	24.1 (18.8–29.3)	22.5 (21.1–23.9)	
South	29.4 (23.8–35.0)	36.9 (35.1–38.6)	
West	25.0 (19.0–31.1)	21.1 (19.5–22.7)	

**Table 2:** Extracted factor characteristics of each study domain following factor analysis.

Study Measure	Factor Loadings	Communality Estimates
**Medical care utilization (in the last 12 months)**	**Factor eigenvalue: 1.30**	
Received care 10+ times	0.59	0.35
>4 days hospitalized, if hospitalized ≥ 1×	0.56	0.31
>1 times hospitalized, if hospitalized ≥ 1×	0.55	0.30
>1 emergency room visit	0.29	0.09
>7 visits to a doctor or health professional	0.47	0.22
Saw or spoke to medical specialist	0.19	0.04
**Financial barriers to care (in the last 12 months)**	**Factor eigenvalue: 4.16**	
Delayed medical care due to cost	0.61	0.37
Delayed refilling medications to save money	0.80	0.63
Took less medication to save money	0.80	0.64
Skipped medications to save money	0.77	0.59
Could not afford medical care	0.68	0.46
Could not afford dental care	0.52	0.27
Could not afford medications	0.71	0.50
Could not afford follow-up care	0.57	0.33
Could not afford specialist care	0.60	0.36
**Financial distress of affording care**	**Factor eigenvalue: 4.41**	
Worried about standard of living	0.76	0.58
Worried about medical costs of illness/accident	0.77	0.59
Worried about paying rent	0.82	0.67
Worried about credit card payments	0.73	0.54
Worried about medical costs of healthcare	0.72	0.52
Worried about money for retirement	0.76	0.58
Worried about monthly bills	0.84	0.71
Worried about medical bills	0.48	0.23

**Table 3:** Multivariable regressions of (**A**) extracted domain factors and blood cancer diagnosis and (**B**) individual outcomes and blood cancer in the domains of medical care utilization, financial barriers to care, and financial distress of affording care, 2014–2020 ^a^.

(A) Association of Extracted Domain Factors and Blood Cancer Diagnosis			
Extracted Factor for Each Domain	Regression Coefficient (β) Estimate	t Value	*p* Value
Medical care utilization (in the last 12 months)	0.36	2.35	0.02
Financial barriers to care (in the last 12 months)	−0.19	−4.84	<0.0001
Financial distress of affording care	0.64	2.28	0.03
**(B) Association of Individual Study Outcomes and Blood Cancer Diagnosis**			
	**Odds ratio (95% CI), compared to** **solid tumor respondents**		**Wald’s *p* value**
**Medical care utilization (in the last 12 months)**			
Received care 10+ times	1.19 (0.84–1.69)		0.34
>4 days hospitalized, if hospitalized ≥ 1×	1.37 (0.90–2.08)		0.15
>1 times hospitalized, if hospitalized ≥ 1×	1.67 (0.97–2.89)		0.07
>1 emergency room visit	0.93 (0.70–1.24)		0.61
>7 visits to a doctor or health professional	1.01 (0.77–1.34)		0.93
Saw or spoke to medical specialist	1.73 (1.17–2.57)		0.01
**Financial barriers to care (in the last 12 months)**			
Delayed medical care due to cost	0.47 (0.22–1.03)		0.06
Delayed refilling medications to save money	0.37 (0.18–0.76)		0.01
Took less medication to save money	0.42 (0.20–0.86)		0.02
Skipped medications to save money	0.49 (0.25–0.99)		0.05
Could not afford medical care	0.52 (0.18–1.53)		0.24
Could not afford dental care	0.87 (0.51–1.49)		0.61
Could not afford medications	0.51 (0.27–0.96)		0.04
Could not afford follow-up care	0.29 (0.10–0.89)		0.03
Could not afford specialist care	0.22 (0.07–0.73)		0.01
**Financial distress of affording care**			
Worried about standard of living	1.25 (0.64–2.44)		0.52
Worried about medical costs of illness/accident	0.86 (0.43–1.71)		0.66
Worried about paying rent	1.58 (0.83–2.99)		0.16
Worried about credit card payments	2.09 (0.62–7.00)		0.23
Worried about medical costs of healthcare	3.36 (1.50–7.51)		<0.01
Worried about money for retirement	1.64 (0.87–3.10)		0.12
Worried about monthly bills	1.23 (0.62–2.45)		0.55
Worried about medical bills	1.30 (0.78–2.17)		0.32

^a^ Multivariable weighted linear (Table 3A) and logistic (Table 3B) adjusting for statistically significant baseline variables, including sex, total combined family income, and baseline health status.

## Data Availability

The data presented are publicly accessible via the Integrated Public Use Microdata Series (https://www.nhis.ipums.org (accessed on 10 January 2022)).

## Supplementary Materials

The following are available online at https://www.mdpi.com/article/10.3390/cancers14071605/s1, Table S1: Cancer types of respondents, Table S2: Description of NHIS survey questions selected as study measures, Figure S1: Factor analysis and extraction for study domains.
