# Supplementary material for: Divergent Patterns in Care Utilization and Financial Distress between Patients with Blood Cancers and Solid Tumors: A National Health Interview Survey Study, 2014–2020
Source: Cancers (Basel). 2022 Mar 22;14(7):1605. doi: 10.3390/cancers14071605 (PMC8996850; doi:10.3390/cancers14071605)
Supplement: Supplementary file 1 [file cancers-14-01605-s001.zip › cancers-1625765-supplementary.pdf]

Divergent Patterns in Care Utilization and Financial Distress between Patients with Blood Cancers and Solid Tumors: A National Health Interview Survey Study, 2014–2020

Christopher T. Su, Christine M. Veenstra and Minal R. Patel

Table S1. Cancer types of respondents.

Type of Cancer	Respondents ≤5 Years Since Diagnosis
**Blood cancers**	**398**
Blood cancer only	50 (13%)
Leukemia only	119 (30%)
Lymphoma only	224 (56%)
2 types of blood cancer	5 (1%)
**Solid tumors**[a]	**6248**
Bladder cancer only	211 (3%)
Bone cancer only	45 (<1%)
Brain cancer only	43 (<1%)
Breast cancer only	1399 (22%)
Cervical cancer only	197 (3%)
Colon cancer only	426 (7%)
Esophagus cancer only	39 (<1%)
Gallbladder cancer only	8 (<1%)
Kidney cancer only	129 (2%)
Larynx cancer only	24 (<1%)
Liver cancer only	63 (1%)
Lung cancer only	353 (6%)
Melanoma only	572 (9%)
Mouth/tongue/lip cancer only	38 (<1%)
Ovarian cancer only	109 (2%)
Pancreatic cancer only	57 (<1%)
Prostate cancer only	1068 (17%)
Rectal cancer only	53 (<1%)
Soft tissue cancer only	26 (<1%)
Stomach cancer only	51 (<1%)
Testicle cancer only	31 (<1%)
Throat cancer only	83 (1%)
Thyroid cancer only	188 (3%)
Uterine cancer only	217 (3%)
2 types of solid tumor	712 (11%)
3 types of solid tumor	106 (2%)

[a] Solid tumor was defined as cancers arising from a non-hematopoietic origin, which included all of the cancer types listed in the NHIS interview guide with the exception of "other," "skin (unspecified)," and "skin (non-melanoma)."

Table S2. Description of NHIS survey questions selected as study measures.

Study Measure Name in Figure 2	IPUMS Variable Name	Variable Type	Study Data Years	Most Recent NHIS Survey Question Text (Within the Study Period)
**Medical care utilization (in the last 12 months)**				
Received care 10+ times	CARE10X	Categorical	2014-2018	During the past 12 months, did you receive care from doctors or other health care professionals 10 or more times? Do not include telephone calls.
>4 days hospitalized, if hospitalized ≥1x	HOSPNITE	Continuous, transformed to categorical based on median (4)	2014-2018	Altogether how many nights were you in the hospital during the past 12 months?
>1 times hospitalized, if hospitalized ≥1x	HOSPNUM	Continuous, transformed to categorical based on median (1)	2014-2018	How many different times did you stay in any hospital overnight or longer during the past 12 months?
>1 emergency room visit	ERYRNO	Continuous, transformed to categorical based on median (1)	2014-2020	During the past 12 months, how many times have you gone to a hospital emergency room about your own health (this includes emergency room visits that resulted in a hospital admission)?
>7 visits to a doctor or health professional	VISITYRNO	Continuous, transformed to categorical based on median (6-7 visits)	2014-2018	During the past 12 months, how many times have you seen a doctor or other health care professional about your own health at a doctor's office, a clinic, or some other place? Do not include times you were hospitalized overnight, visits to hospital emergency rooms, home visits, dental visits, or telephone calls.
Saw or spoke to medical specialist	SAWSPEC	Categorical	2014-2018	During the past 12 months, have you seen or talked to a medical doctor who specializes in a particular medical disease or problem (other than obstetrician/gynecologist, psychiatrist, or ophthalmologist) about your own health?
**Financial barriers to care (in the last 12 months)**				
Delayed medical care due to cost	DELAYCOST	Categorical	2014-2020	During the past 12 months, have you delayed seeking medical care because of worry about the cost?
Delayed refilling medications to save money	YDELAYMEDYR	Categorical	2014-2020	During the past 12 months, you delayed filling a prescription to save money.
Took less medication to save money	YSKIMPMEDYR	Categorical	2014-2020	During the past 12 months, you took less medicine to save money.
Skipped medications to save money	YSKIPMEDYR	Categorical	2014-2020	During the past 12 months, you skipped medication doses to save money.
Could not afford medical care	YBARCARE	Categorical	2014-2018	During the past 12 months, was there any time when you needed medical care, but did not get it because you couldn't afford it?
Could not afford dental care	YBARDENTAL	Categorical	2014-2018	During the past 12 months, was there any time when you needed dental care (including check ups), but didn't get it because you couldn't afford it?
Could not afford medications	YBARMEDS	Categorical	2014-2020	During the past 12 months, was there any time when you needed prescription medicines, but didn't get it because you couldn't afford it?
Could not afford follow-up care	YBARFOLLOW	Categorical	2014-2017	During the past 12 months, was there any time when you needed follow-up care, but didn't get it because you couldn't afford it?
Could not afford specialist care	YBARSPECL	Categorical	2014-2017	During the past 12 months, was there any time when you needed to see a specialist, but didn't get it because you couldn't afford it?
**Financial distress of affording care**				
Worried about standard of living	WRYSTDLIV	Categorical	2014-2018	How worried are you right now about not being able to maintain the standard of living you enjoy?
Worried about medical costs of illness/accident	WRYMEDCST	Categorical	2014-2018	How worried are you right now about not being able to pay medical costs of a serious illness or accident?
Worried about paying rent	WRYHOUS	Categorical	2014-2018	How worried are you right now about not being able to pay your rent, mortgage, or other housing costs?
Worried about credit card payments	WRYCCPAY	Categorical	2014-2018	How worried are you right now about not being able to make the minimum payments on your credit cards?
Worried about medical costs of healthcare	WRYHCCST	Categorical	2014-2018	How worried are you right now about not being able to pay medical costs for normal healthcare?
Worried about money for retirement	WRYRET	Categorical	2014-2018	How worried are you right now about not having enough money for retirement?
Worried about monthly bills	WRYBILLS	Categorical	2014-2018	How worried are you right now about not having enough to pay your normal monthly bills?
Worried about medical bills	WORMEDBILL	Categorical	2014-2020	If you get sick or have an accident, how worried are you that you will be able to pay your medical bills?

(A) **Medical care utilization**

Eigenvalues of the Reduced Correlation Matrix: Total = 1.35669937 Average = 0.22611656				
	Eigenvalue	Difference	Proportion	Cumulative
1	1.30047743	0.68576675	0.9586	0.9586
2	0.61471068	0.58447233	0.4531	1.4117
3	0.03023834	0.15789338	0.0223	1.4339
4	-.12765504	0.08630387	-0.0941	1.3398
5	-.21395891	0.03315424	-0.1577	1.1821
6	-.24711314		-0.1821	1.0000

1 factor will be retained by the NFACTOR criterion.

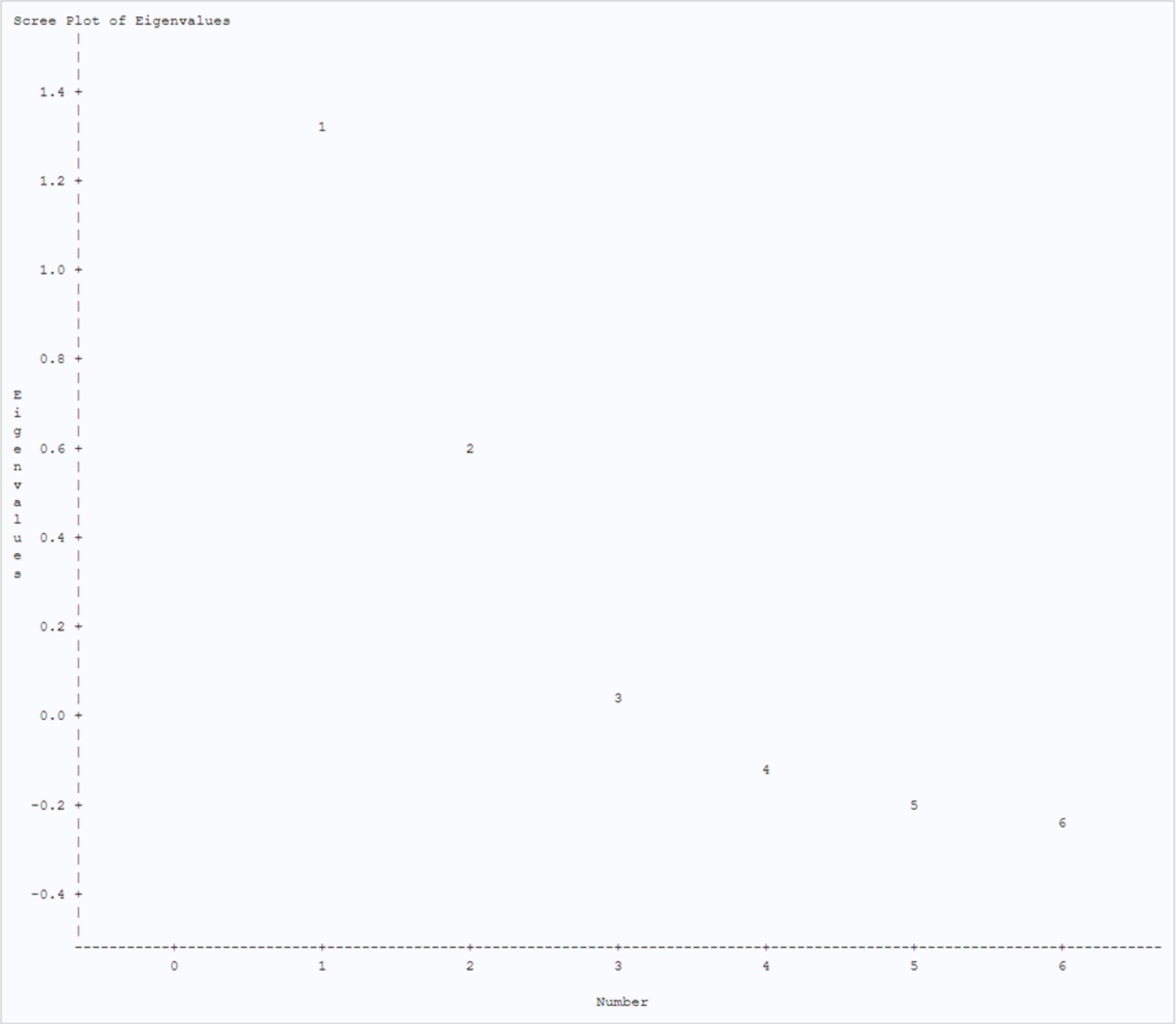

(B) **Financial barriers to care**

Eigenvalues of the Reduced Correlation Matrix: Total = 4.74789902 Average = 0.52754434				
	Eigenvalue	Difference	Proportion	Cumulative
1	4.16435326	3.42661533	0.8771	0.8771
2	0.73773793	0.34581688	0.1554	1.0325
3	0.39192106	0.32076189	0.0825	1.1150
4	0.07115917	0.11818701	0.0150	1.1300
5	-.04702784	0.03417101	-0.0099	1.1201
6	-.08119885	0.03649531	-0.0171	1.1030
7	-.11769416	0.04977308	-0.0248	1.0782
8	-.16746724	0.03641707	-0.0353	1.0429
9	-.20388431		-0.0429	1.0000

1 factor will be retained by the NFACTOR criterion.

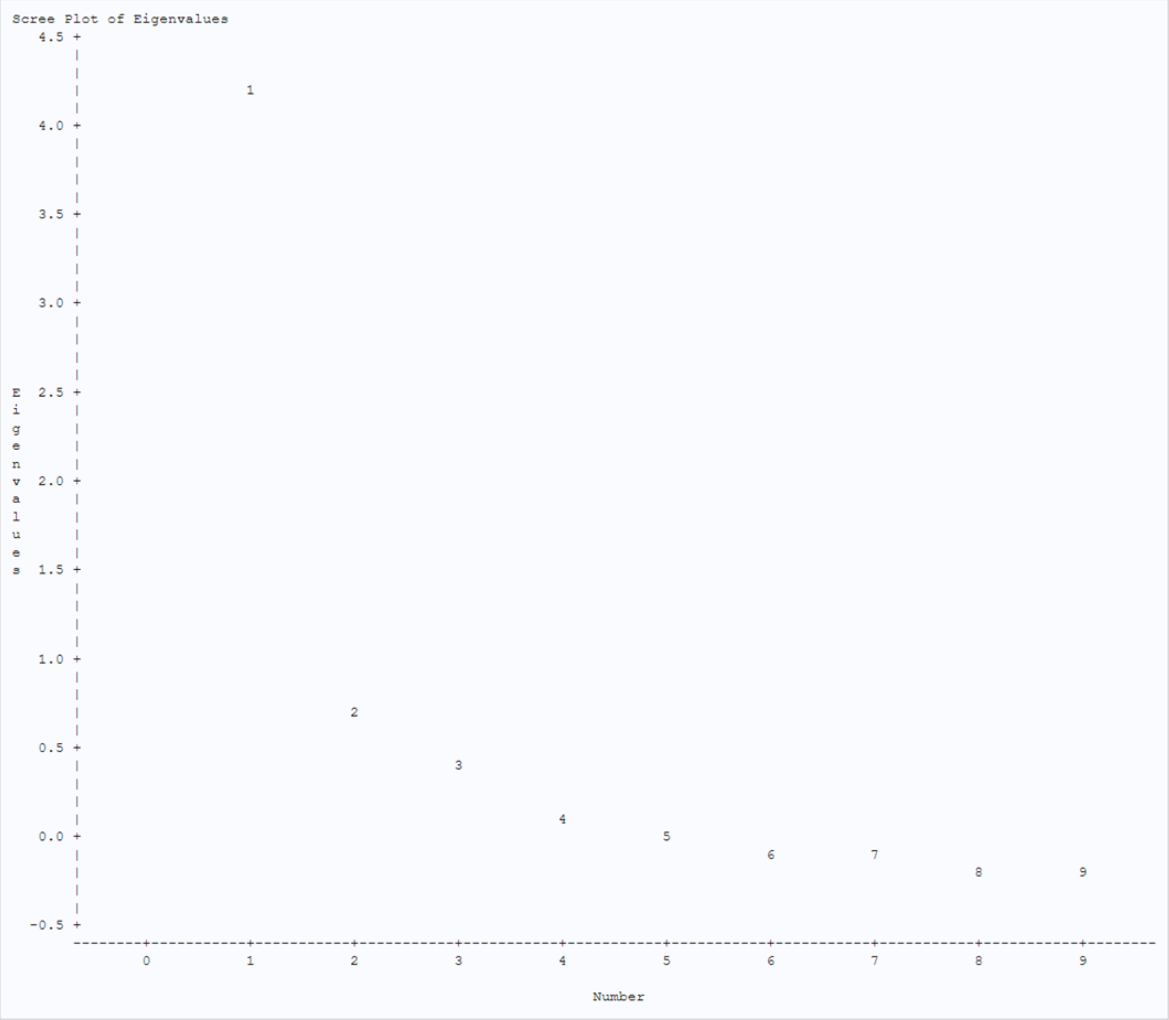

(C) **Financial distress of affording care**

Eigenvalues of the Reduced Correlation Matrix: Total = 4.82490128 Average = 0.60311266				
	Eigenvalue	Difference	Proportion	Cumulative
1	4.41435468	3.75220688	0.9149	0.9149
2	0.66214780	0.54472397	0.1372	1.0521
3	0.11742383	0.05044101	0.0243	1.0765
4	0.06698282	0.12553951	0.0139	1.0904
5	-.05855669	0.02884915	-0.0121	1.0782
6	-.08740584	0.03303623	-0.0181	1.0601
7	-.12044207	0.04916120	-0.0250	1.0352
8	-.16960327		-0.0352	1.0000

1 factor will be retained by the NFACTOR criterion.

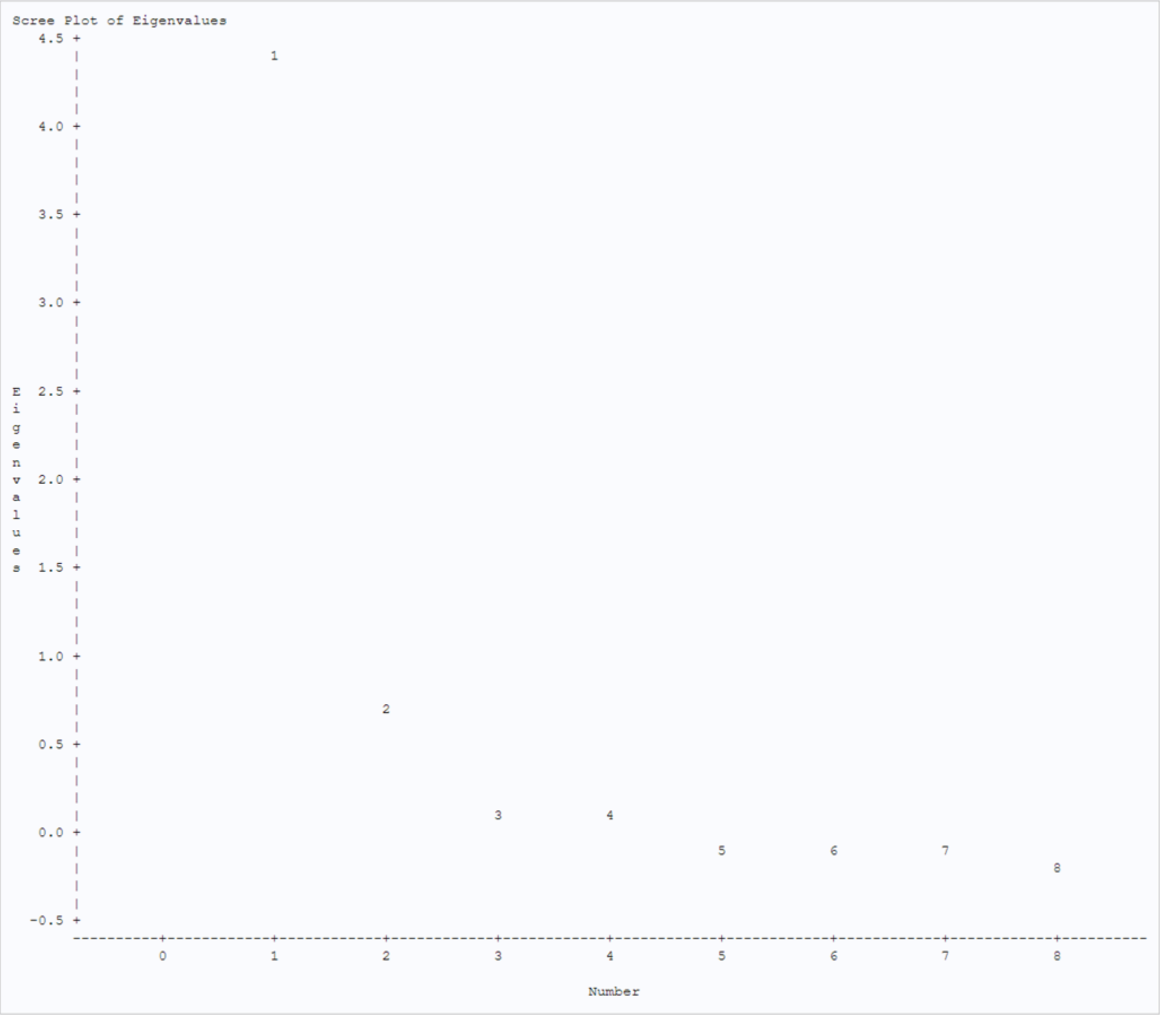

Figure S1. Factor analysis and extraction for the study domains of (**A**) medical care utilization, (**B**) financial barriers to care, and (**C**) financial distress of affording care. The scree plot visually represents the eigenvalues of each identified factor in the analysis. As a result, one factor meeting the Kaiser criterion (with eigenvalue >1) was retained for each study domain as the representative factor for the domain.
